# Multiple roles of dihomo-γ-linolenic acid against proliferation diseases

**DOI:** 10.1186/1476-511X-11-25

**Published:** 2012-02-14

**Authors:** Xiaoping Wang, Huanping Lin, Yan Gu

**Affiliations:** 1 Laboratory of Molecular Pathology, Shaanxi University of Chinese Medicine, Xianyang 712046, Shaanxi, China; 2 Department of Pharmaceutical Sciences, North Dakota State University, Fargo 58102, ND, USA

**Keywords:** Dihomo-γ-linolenic acid (DGLA), Prostaglandin E1 (PGE1), Proliferation, Differentiation, Cancer

## Abstract

Considerable arguments remain regarding the diverse biological activities of polyunsaturated fatty acids (PUFA). One of the most interesting but controversial dietary approaches focused on the diverse function of dihomo-dietary γ-linolenic acid (DGLA) in anti-inflammation and anti-proliferation diseases, especially for cancers. This strategy is based on the ability of DGLA to interfere in cellular lipid metabolism and eicosanoid (cyclooxygenase and lipoxygenase) biosynthesis. Subsequently, DGLA can be further converted by inflammatory cells to 15-(S)-hydroxy-8,11,13-eicosatrienoic acid and prostaglandin E_1 _(PGE_1_). This is noteworthy because these compounds possess both anti-inflammatory and anti-proliferative properties. PGE1 could also induce growth inhibition and differentiation of cancer cells. Although the mechanism of DGLA has not yet been elucidated, it is significant to anticipate the antitumor potential benefits from DGLA.

## 

Supplementation of cell cultures in vitro or feeding animals with ω-3 or ω-6 polyunsaturated fatty acids (PUFAs) led to an increase of these PUFAs in cell membrane phospholipids and may influence membrane properties [1,2]. Moreover, PUFAs and their metabolites, eicosanoids, are considered as important mediators and modulators of the intracellular network of signals [3], they change oxidative metabolism and may have a direct effect on gene expression when activating the specific nuclear receptors and transcription factors [3-5]. ω-3 types PUFAs were reported to improve immunological response, prevent proliferation and initiate apoptosis, kill tumor cells in vitro [6-8], and inhibit tumor growth in experimental animals, while ω-6 PUFAs are supposed not to possess such significant antitumor effects even generate opposite action [9,10].

One of the most interesting but controversial dietary approaches focused on the diverse function of dihomo-dietary γ-linolenic acid (DGLA) in anti-inflammation and anti-proliferation diseases although it is a distinct ω-6 type PUFA [11-13]. To evaluate DGLA-related nutraceuticals critically, it is essential to elucidate the mechanisms underlying the relationship between DGLA and health maintenance. This review will focus on recent studies that address the physiologic functions and mechanisms of function of DGLA in proliferation and hyperplasia diseases.

Dihomo-γ-linolenic acid (DGLA) is a 20-carbon ω-6 polyunsaturated fatty acid (PUFA) derived in vivo from linolenic acid, an essential fatty acid. DGLA can then be converted to arachidonic acid (AA), another 20-carbon ω-6 PUFA [11,12]. Both DGLA and AA are substrates of the lipid-peroxidizing enzyme COX. Through a series of free radical reactions, COX metabolizes DGLA and AA to form various bioactive metabolites, namely, the 1 and the 2 series of prostaglandins (PGs1 and PGs2), respectively. Unlike PGs2, which are generally viewed as pro-inflammatory, PGs1 actually possess anti-inflammatory and anticancer activities [14-16]. For example, PGE_1_, one form of PGs1, could inhibit vascular smooth muscle cell proliferation, reduce vascular cell adhesion, and attenuate the development of atherosclerosis [17-19].

### DGLA metabolism

It is generally thought that all mammals, including humans, require 1-2% of total dietary energy as linoleic acid [LA, 18:2(n-6)] to prevent essential fatty acid deficiency [12]. LA is metabolized in a variety of tissues by Δ6 desaturase to form GLA, which is rapidly elongated to DGLA (Figure 1). DGLA can be further desaturated to arachidonic acid [AA, 20:4(n-6)] by Δ5 desaturase. However, due to the limited activity of Δ5 desaturase in rodents and humans, only a partial DGLA is converted to AA [20,21]. These data indicate that in many cell types, DGLA, the elongase product of GLA, but not AA, accumulates after GLA supplementation.

**Figure 1:**
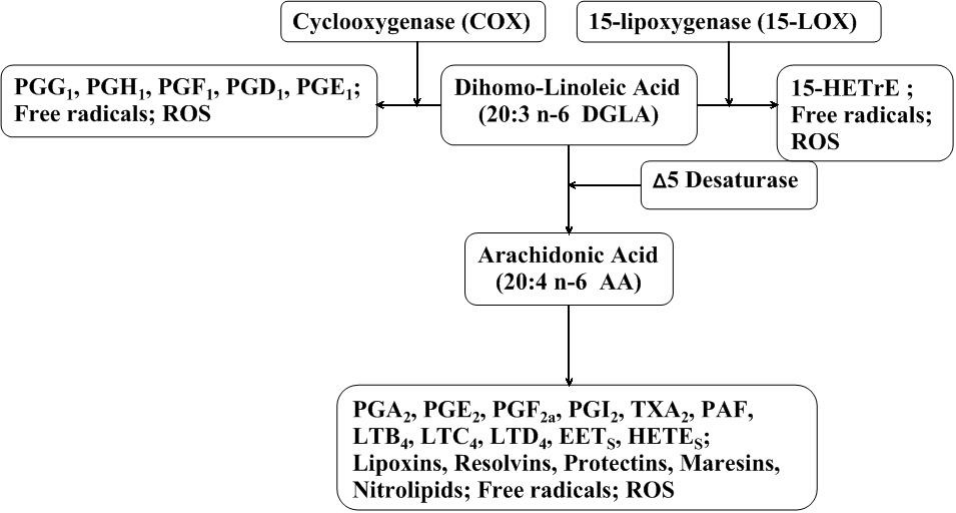
**Products of dihomo-γ-linolenic acid**. In mammal tissues and cells, DGLA is converted to AA by an alternating sequence of Δ5 desaturation. DGLA can be converted to PG_1 _via the cyclooxygenase pathway and/or converted to 15-HETrE via the 15-lipoxygenase pathway. During the process of conversion mediated by different oxygenases, free radicals and lipid perioxidation and various metabolites were generated.

The increase in DGLA relative to AA is able to attenuate the biosynthesis of AA metabolites, i.e., 2-series prostaglandins, 4-series leukotrienes and platelet-activating factor (PAF), and exerts an anti-inflammatory effect in human subjects [20-22] (Figure 1). Another research demonstrated that addition of GLA or DGLA together with a mixed D5/D6 fatty-acid desaturase inhibitor CP-24879 inhibited D5 desaturase activity and the further conversion of DGLA into AA. This led to a very substantial increase in the accumulation of DGLA from 2.3% to almost 12% of total fatty acids without a change in the level of AA [16].

In addition, because addition of DGLA bypasses a key regulatory rate-limiting enzymatic step (Δ6 desaturase) which controls the formation of long-chain PUFA of the (ω-6) series, it may generate a systemic decline in Δ6 desaturation. Researches has confirmed that a reduced capacity to convert LA to DGLA has been associated with various physiologic and pathophysiologic states, including aging, diabetes, alcoholism, atopic dermatitis, premenstrual syndrome, rheumatoid arthritis, cancer and cardiovascular disease [12,17,23]. Therefore, supplementation of DGLA may be of value in alleviating some of the symptoms of these various diseases.

### DGLA metabolic oxidation and mechanisms against proliferation diseases

Alteration in the dietary content of fatty acids can lead to modulation of the structure/function of membrane-bound receptors, cell-cell interaction, enzyme activities, cellular signalling and eicosanoid production [24,25]. Eicosanoid synthesis is dependent on the size of the fatty acid precursor pool(s) and on the availability of substrate fatty acids released from phospholipids [26,27]. Depending on the cell types, DGLA is cyclooxygenated (by COX-1/2) to prostaglandins of the 1-series (PGE_1_) and/or metabolized by the 15-lipoxygenase into 15-(S)-hydroxy-8,11,13-eicosatrienoic acid (15-HETrE) [28] (Figure 1, 2). These two oxidative metabolites of DGLA, have been found to exert clinical efficacy in a variety of diseases, including suppression of chronic inflammation, vasodilation and lowering of blood pressure, inhibition of smooth muscle cell proliferation associated with atherosclerotic plaque development, arresting of cancer cell growth and the differentiation of tumor cells [15,29-31].

**Figure 2:**
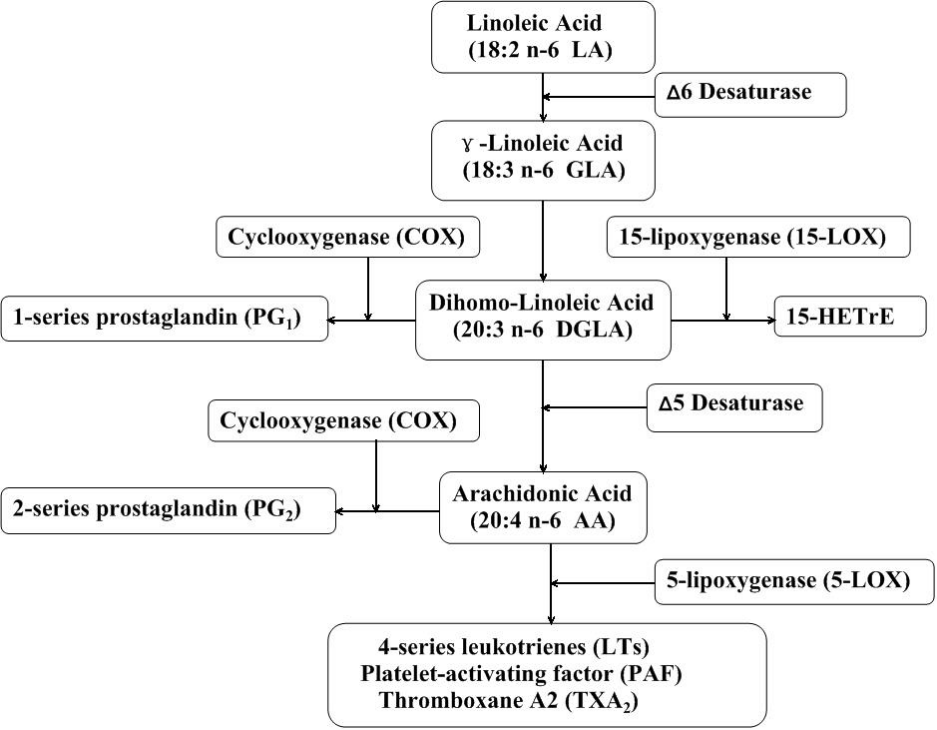
**Metabolism of dihomo-γ-linolenic acid**. In mammal tissues and cells, LA is converted to AA by an alternating sequence of Δ6 desaturation, chain elongation and Δ5 desaturation. Dietary GLA bypasses the rate-limited Δ6 desaturation step and is quickly elongated to DGLA by elongase, with only a very limited amount being desaturated to AA by Δ5 desaturase. DGLA can be converted to PGE_1 _via the cyclooxygenase pathway and/or converted to 15-HETrE via the 15-lipoxygenase pathway.

COX-2, a rate-limiting enzyme in the pathway of PG synthesis, is one of the interesting cellular factors and has been suggested to be associated with carcinogenesisin colorectal cancer [32-34]. The contribution of COX-2 to tumorigenesis includes an increased resistance to apoptotic stimuli, enhanced invasiveness, and stimulated angiogenesis [14,32-35]. In addition, expression of COX-2 has also been found to be high in other types of cancers including prostate, breast, gastric, pancreatic, liver, lung, and skin cancers [36-42]. COX enzymes are integralmembrane proteins located in the endoplasmic reticulumand nuclear membrane. These proteins are known to catalyze the rate-limiting step in the metabolism of arachidonic acid (AA), resulting in the production of prostaglandin G2 (cyclooxygenase reaction) [16,43]. Prostaglandin G2 is then converted to prostaglandin H2 (peroxidase reaction), a target of several specific prostanoid synthases, resulting in the production of prostaglandins, prostacyclins, and thromboxanes [43,44]. Two COX isoforms have been identified. COX-1 is constitutively expressed in most normal mammalian tissues,where it is involved in the maintenance of tissue homeostasis.In contrast, COX-2 is rapidly expressed as a consequence of stimulation by growth factors, cytokines, inflammatory mediators, and tumor promoters [44,45]. While a paradoxical aspect of COX-2 is that it could also convert DGLA to PGE_1 _which possesses anti-inflammation, anti-proliferation and promoting differentiation capacity [45]. It seemed that the ratio of PGE_1_/PGE_2 _mediated by COX-2 produced a balance efficancy on cell growth.

The majority of prostaglandins, including PGE_1_, PGE_2_, PGI_2_, PGD_2_, PGF_2_, and TXA_2_, are quickly secreted from cells and bind locally to membrane-bound prostanoid receptors termed EP, IP, DP, FP, and TP, respectively. The EP receptor subclass is further divided into four subtypes EP1, EP2, EP3, and EP4 [46]. With regard to tumor biology, the prostanoid PGE_2 _stimulates the growth and invasion of several different tumor cells and promotes angiogenesis by increasing VEGF production [47-49]. In comparison, PGE_1 _possesses distinct anti-inflammation and anti-proliferation action [15,50]. GLA or DGLA supplementation studies conducted in humans and rodents have shown that the synthesis of 1-series prostaglandins, and not the 2-series prostaglandins (PGE_2_, derived from AA), is selectively elevated [16,17]. Although the increases in the tissue levels of PGE_1 _after DGLA supplementation are modest relative to PGE_2_, effects are noteworthy because select biological properties of PGE_1 _are ~20 times stronger than PGE_2 _[51]. In particular, PGE_1 _elicits an array of intracellular responses by binding to select G protein coupled surface PGE (EP) receptors and/or the prostacyclin (IP) receptor [52]. Four subtypes of the EP-receptor, termed EP_1_, EP_2_, EP_3 _and EP_4 _have been identified. The EP_2_, EP_4 _and IP receptors couple to adenylatecyclase via a G_s_-protein, and receptor activation results in increases in intracellular levels of cyclic 3',5'-adenosine monophosphate (cAMP) [52,53]. This in turn stimulates the expression of numerous genes through the PKA-mediated phosphorylation of the nuclear CREB binding proteins. The transcriptional co-activator, CBP, in turn, mediates PKA-induced transcription by binding to the PKA phosphorylated activation domain of CREB. CREB proteins can also heterodimerize with other members of the b-ZIP or basic zipper family of transcription factors, including Fos proteins (c-fos, Fosb, Fra-1, Fra-2), and Jun proteins (c-jun, JunB, JunD). Elevation of cAMP stimulates the expression of numerous genes through the protein kinase A (PKA)-mediated phosphorylation of the nuclear cAMP response element binding proteins (CREB). Through this mechanism, PGE_1 _has been shown to inhibit vascular smooth muscle cell (SMC) proliferation in vitro [53,54]. It is significant because reducing the migration and proliferation of vascular SMC could arrest the formation of the typical atherosclerotic plaque [55,56]. The question of whether PGE_1 _and PGE_2 _act on the same or different receptors is not yet resolved. Some reports provide data to demonstrate that PGE_1 _binds to the PGE_2 _receptors EP2 and EP4 [57,58], indicating that PGE_1 _could compete with PGE_2 _through binding to the same receptors.

More encouraging researches indicate that several diseases may benefit from GLA or DGLA administration [12,17,20-22]. It is reported that GLA or DGLA suppresses human synovial cell proliferation in culture by increasing PGE_1 _synthesis and intracellular cAMP levels [59]. In addition, administration of GLA or DGLA is capable of suppressing human T-cell proliferation [12,60]. Although the precise mechanism of responses remains to be investigated, it has been suggested that the incorporation of select diet-derived PUFA may alter the dynamic lipid environment that influences protein lateral diffusion of anchored receptors, thereby modulating their function [2,3]. These findings highlight the need for establishing a therapeutic GLA or DGLA dose that will down-regulate autoimmune and cell-mediated responses.

Detection of the oxidative metabolism of DGLA into lipoxygenase products has shown that some cell types, including neutrophils, macrophage/monocytes and epidermal cells, metabolize DGLA into the 15-lipoxygenase product, 15-HETrE. Researches suggest that 15-lipoxygenase-derived hydroxy fatty acids inhibit the synthesis of AA-derived 5-lipoxygenase metabolites [11,12,28] (Figure 3). The observations are significant because elevated levels of AA-derived 5-lipoxygenase products, e.g., LTC_4 _and LTB_4_, are associated with several pathologic inflammatory, hyperproliferative disorders [61,62]. Studies also indicate that 15-HETrE can be incorporated into the membrane phospholipid, phosphatidylinositol 4,5-bisphosphate (PtdIns 4,5-P_2_), and released as 15-HETrE-containing-diacylglycerol (15-HETrE-DAG) [63,64]. Interestingly, 15-HETrE-DAG is able to inhibit protein kinase C β (PKC β), a mediator of the cell cycle in select cell types [63]. The inhibitions of leukotriene biosynthesis and PKC-dependent signal transduction are probable mechanisms by which DGLA exertsanti-inflammation and anti-proliferative responses. It is generally thought that DGLA-derived eicosanoid (PGE_1 _and 15-HETrE) biosynthesis is dependent primarily on the abundance of nonesterified DGLA, so increasing the administration of DGLA may be a good strategy to treat some proliferation diseases.

**Figure 3:**
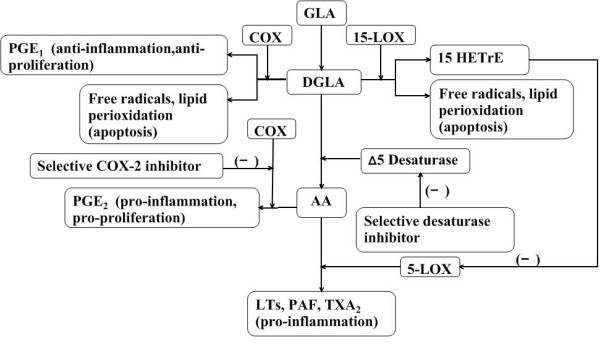
**Mechanisms of dihomo-γ-linolenic acid in anti-proliferation of diseases**. DGLA-derived PGE_1 _has been identified as possessing anti-inflammatory properties that differentiate it from AA-derived PGE_2_. DGLA could be metabolized into the 15-lipoxygenase product, 15-HETrE, which is capable of inhibiting the synthesis of AA-derived 5-lipoxygenase metabolites and further attenuates the pro-inflammatory products from AA. All types of free radicals (superoxide anion, H2O2, hydroxyl radicals) and lipid peroxides play a role in the induction of apoptosis of tumor cells by the metabolism of DGLA. Selective COX-2 inhibitor could stop AA from converting to PGE_2 _which are able to stimulate cancer cell proliferation. DGLA may be accumulated through blocking the conversion to AA mediated by selective desaturase inhibitor.

### Efficacy of DGLA against tumors

A growing number of studies suggest that DGLA is unique among the (n-6) PUFA family members (LA, GLA and AA) in its potential to suppress tumor growth and metastasis. DGLA has the ability to inhibit both motility and invasiveness of human colon cancer cells by increasing the expression of E-cadherin, a cell-to-cell adhesion molecule that acts as a suppressor of metastasis [65,66]. In addition, DGLA reduces tumor-endothelium adhesion, a key factor in the establishment of distant metastases, partly by improving gap junction communication within the endothelium [65,67]. These encouraging results indicate that further investigations should be given priority.

Since PUFAs enhance free radical generation and lipid peroxidation process and thus, induce apoptosis, it suggests that these events lead to depletion of ATP levels in the tumor cells which brings about their death. This implies that an interaction exists among fatty acids, lipid peroxidation process, apoptosis and genes/oncogenes that regulate apoptotic process [6-10]. Researches previously have showed that some polyunsaturated fatty acids (PUFAs) induced apoptosis of tumor cells with little or no cytotoxic action on normal cells under certain conditions [6,68,69]. It was observed that of all the fatty acids tested, GLA or DGLA was the most effective in selectively killing the tumor cells [6]. In a co-culture experiment wherein normal human skin fibroblasts and human breast cancer cells were grown together in a petri dish supplemented with GLA or DGLA, only human breast cancer cells were eliminated without any effect on normal skin fibroblasts [65,68,70,71]. These results reconfirmed that GLA or DGLA show selective tumoricidal action at least in vitro. These molecular changes were found to be significantly associated with enhanced degree of lipid peroxidation in the fatty acid supplemented tumor cells [6,65,68-71]. GLA and DGLA were also found to be capable of suppressing the expression of oncogenes Her-2/neu and Bcl-2 and enhance p53 activity and thus, induce apoptosis of tumor cells [72-74]. In an extension of these studies, it was noted that cyclooxygenase and lipoxygenase inhibitors blocked the tumoricidal action of DGLA on human cervical carcinoma; whereas anti-oxidants inhibited cytotoxic action of DGLA on human breast cancer cells [6,73,75-77]. Prostaglandins (PGE_1_) from DGLA mediated by COX inhibited the growth of HeLa cells [77,78]. LOX products were more potent than PGs in inhibiting of HeLa cell growth which was confirmed by the observation that a 9-fold increased formation of hydroxides occurred in HeLa cells [77]. These results suggest that both COX products, LOX products and free radicals, lipid peroxidation are involved in the tumoricidal action of DGLA. A significant increase in the formation of free radicals and lipid peroxides was noted only in tumor cells treated with DGLA compared to untreated tumor cells [6-10,75-78], suggesting that the involvement of COX and LOX products, free radicals and lipid peroxides in the tumoricidal action of DGLA varies depending on the cell type that is being tested. Recent studies demonstrated that the longer-chain n-6 PUFA produced by GLA feeding decreased the Th2 cytokine and immunoglobulin (Ig)G1 antibody response. GLA and DGLA could also induce T-regulatory cell activity, e.g., transforming growth factor (TGF)-beta-producing T cells, and reduce proinflammatory interleukin (IL)-1 and tumor necrosis factor (TNF)-alpha production [79], indicating immunity mechanism is likely to participate in the anti-tumor effect.

Drug resistance is a major issue in the management of cancer. Hence, methods or strategies to prevent and reverse tumor cell drug resistance are needed. More studies confirmed that GLA and DGLA could kill drug resistant tumor cells in vitro and augment tumoricidal actions of anti-cancer drugs synergistically [6,65,68-71]. But, it is not clear as to the exact mechanism by which this synergism between anti-cancer drugs and DGLA occurs. The possible mechanism lies in DGLA could augment uptake and decrease efflux of anti-cancer drugs and thus, reverse tumor cell drug resistance [6,75].

However, the mechanism of action mediating DGLA effects was not fully elucidated, because feeding of GLA or DGLA as ethyl esters or triglycerides (triacylglycerols) generally led to only a small increase in GLA or DGLA content in cell membrane lipids, often accompanied by a very significant increase in AA content. Furthermore, the ratio of PGE_1_/PGE_2 _produced in cells obtained from animals fed with GLA-containing oils was shown to be substantially lower than the cellular ratio of DGLA/AA [16,17]. Similarly, in studies with cultured mouse fibrosarcoma cells rendered deficient in essential fatty acids and then replenished with either DGLA or AA, the ratio of synthesized PGE_1_/PGE_2 _was considerably smaller than the cellular ratio of DGLA/AA [80,81]. It should, however, be pointed out that in both in vivo dietary study and in vitro cell culture study, the cellular content of AA, even after significant enrichment with DGLA, was still 2.5 ± 3-fold higher than DGLA, mainly due to effective desaturation of DGLA to AA [16,81,82]. Therefore, an effective solution to accumulate the content of DGLA is to administer selective Δ5 desaturase inhibitors to stop DGLA from further converting to AA. Sesame, curcumin and other analog desaturase inhibitors, for example, CP-24879, a d5/d6 desaturase inhibitor, have been verified to exert anti-proliferation or anti-cancer effect by enhancing the concentration of DGLA [16,82-84].

While studies showed in some cancer cells, after using the COX and LOX inhibitor, GLA or DGLA only led to a partially growth inhibition of cancer cells, which indicates that the change ratio of PGE_1_/PGE_2 _derived from DGLA metabolites couldn't completely account for the antitumor effect [6,12,16,17]. Recent research has verified that free radicals and lipid perioxidation play an important role in the cytotoxic action to cancer, which could be completely arrested by anti-oxidants such as vitamin E and SOD depending on different cell lines [6]. On the other hand, some reports assume that vitamin E only partially arrests the growth inhibition of cancer cells mediated by PUFAs [70,73,85]. One of the reasons for the different experimental results may originate from various employed cell lines. Another reason is that near all the cytotoxicity effects against cancer cells depend on the time and concentrition of the metabolic products from PUFA. Therefore, DGLA is likely to exert its antitumor cytotoxic effects from free radicals and lipid perioxidation and metabolites, which probably inhibit the proliferation, promote apoptosis and even make cancer cell differentiate and maturation [6-10] (Figure 3). Further investigation need to confirm the presumption.

### Possible adverse effects of oral supplementation with DGLA

A previous report noted that administration of DGLA ethyl ester to health volunteer (1 g/d) reduced platelet aggregation [86]. However, DGLA ethyl ester is an experimental reagent and is not available for practical and nutritional usage. On January 27, 1993, the United States Court of Appeals for the Seventh Circuit ruled that GLA, containing oil is a single food ingredient and therefore not subject to food additive regulation. As a result of this legislation, GLA-containing oils (primrose oil, blackcurrant seed oil and borage oil) have become increasingly popular with retailers and are being sold as encapsulated supplements [17]. To evaluate DGLA-related nutraceuticals critically, it is essential to elucidate the mechanisms underlying the relationship between DGLA and health maintenance. Recent researches indicated that from rodent to human, oral supplementation of DGLA-enriched oil showed no-observed-adverse-effect. In acute and subchronic oral toxicity tests in rats, DGLA oil, ranging from 500, 1000, 2000 mg/kg to 10 g/kg was orally administered. There was no death in either sex and no toxicological changes in body weight, food consumption, ophthalmological examination, urinalysis, hematological examination, blood biochemical examination, necropsy, organ weight, or histopathological examination [87]. The researcher also compared the dietary effects of DGLA with GLA on the fatty acid composition. The resulted showed that the DGLA concentrations in the liver, serum, and brain of the DGLA group were higher than those of the GLA oil group. The DGLA levels in the liver, serum, and brain significantly increased with increasing dosage of DGLA in the diet. DGLA administration significantly increased the ratio of PGE1/PGE2 in the rat plasma. These results suggest that the dietary effect of DGLA would be more dominant than GLA [88]. Further research confirmed the safety of consumption of DGLA in human. DGLA-enriched oil (50 or 150 mg as free DGLA) was administered to healthy men for 4 weeks. The DGLA content in serum phospholipids dose-dependently increased and returned to the initial level after a 4-week washout. No side effects or changes in platelet aggregation were observed. These results indicate that oral supplementation with DGLA oil can safely increase serum DGLA content [89]. Undoubtedly, elucidation of the mechanism(s) of DGLA will lead to the establishment of dietary guidelines designed to reduce the incidence and severity of inflammatory or hyperproliferative diseases.

## Conclusions

In summary, regulation of DGLA administration may have multiple biological benefits for a series of diseases associated with proliferation. DGLA exert anti-proliferation action through enhancing free radicals, lipid perioxidation, PGE_1 _and 15-HETrE synthesis, which directly or indirectly influence the cell growth, apoptosis and differentiation mediated by downstream signal transduction cascades. The consumption of GLA or administration of DGLA may offer new strategies for treatment and prevention of proliferation and hyperplasia diseases.

## Abbreviations

AA: Arachidonic acid; CREB: cAMP Response element binding proteins; DGLA: Dihomo-γ-linolenic acid; EP: PGE Receptor; GLA: γ-linolenic acid; IP: Prostacyclin receptor; LA: Linoleic acid; 15-HETrE: 15-(S)-hydroxy-8,11,13-eicosatrienoic acid; PAF: Platelet activating factor; PGE_1_: Prostaglandin E_1_; ROS: Reactive oxygen species; PKA: Protein kinase A; PLA_2_: Phospholipase A_2_; PUFA: Polyunsaturated fatty acids; SMC: Smooth muscle cell.

## Competing interests

The authors declare that they have no competing interests.

## Authors' contributions

XPW proposed the idea, analyzed the data and wrote the manuscript. HPL and YG collected and interpreted the data. All authors read and approved the final manuscript.
